# Complete Genome Sequence of a Papillomavirus Isolated from the European Mole

**DOI:** 10.1128/genomeA.00530-13

**Published:** 2013-08-01

**Authors:** Piet Maes, Steven Sijmons, Hans Stevens, Marc Van Ranst

**Affiliations:** Laboratory of Clinical Virology, Rega Institute for Medical Research, KU Leuven, Leuven, Belgium

## Abstract

A papillomavirus was isolated from healthy epithelial tissue of two European moles (*Talpa europaea*) and the complete genomic sequence was determined. To our knowledge, this is the first papillomavirus to be isolated from a mole. Phylogenetic analysis shows it to be most closely related to viruses of the genus *Kappapapillomavirus*.

## GENOME ANNOUNCEMENT

The *Papillomaviridae* are a large family of circular, double-stranded epitheliotropic DNA viruses that can cause benign and malignant proliferations of the stratified squamous epithelium. They have been found in a large number of vertebrate species, including man, and are assumed to have evolved alongside their hosts (1). The current virus taxonomy of the International Committee on Taxonomy of Viruses divides the family *Papillomaviridae* into 29 genera, *Alphapapillomavirus* through *Dyoiotapapillomavirus* (2). Novel papillomaviruses are believed to descend from the slow accumulation of point mutations, and different ancient papillomavirus lineages have possibly coevolved and cospeciated with their vertebrate host species (3). Previously reported studies have shown that papillomaviruses are commonly present in the healthy skin of humans and many different animal species (4, 5). We report the discovery and characterization of a novel and distinct mole papillomavirus, the *Talpa europaea* papillomavirus 1 (TePV1), which we isolated from epithelial tissue samples from apparently healthy animals by use of the Illumina MiSeq next-generation sequencing platform.

Epithelial tissue samples (fur and forepaws) from 38 European moles (*Talpa europaea*, Linnaeus, 1758) caught in the wide proximity of Bruges, Belgium, were subjected to whole-genome DNA extraction, and papillomavirus episomal DNA was amplified by multiply primed rolling-circle amplification (6). After purification, the rolling-circle products were subjected to Illumina MiSeq sequencing (Illumina). *De novo* assemblies were generated using CLC Genomics Workbench v5.5.1. Both paired and unpaired reads were coassembled, with a paired read distance minimum/maximum at 180/380. The Sanger sequencing method was used to verify the sequence data acquired by Illumina MiSeq. Sanger sequencing was performed on an ABI Prism 3130xl Genetic Analyzer (Applied Biosystems). The isolation of the virus from two different moles and the historical observation that papillomaviruses are highly species specific suggests that an appropriate name for this virus is *Talpa europaea* papillomavirus 1 (TePV1).

The TePV1 genome has a size of 7,533 bp with a G+C content of 39.10%. The genome organization is typical for papillomaviruses, including five “early” open reading frames, E1, E2, E4, E6, and E7, and two “late” regions, L1 and L2, all located on the same strand of its double-stranded genome. In addition to the classical noncoding region between the early and late regions, a second noncoding region (NCR-2) is present between the end of E2 and the start of L2, which is not typical for the genus *Kappapapillomavirus* but is identified in all members of the genus *Lambdapapillomavirus* (7). Classification of papillomaviruses is based on sequence identity of the L1 gene (2). TePV1 shares L1 nucleotide sequence identities of 67.4%, 64.0%, and 63.1% with, respectively, canine papillomavirus 1 (CPV1), *Oryctolagus cuniculus* papillomavirus 1 (OcPV1), and *Sylvilagus floridanus* papillomavirus 1 (SfPV1). Maximum likelihood analysis showed a close relationship with both OcPV1 and SfPV1, and TePV1 can therefore be considered a tentative new species of the genus *Kappapapillomavirus*, although the other members of this genus lack the additional NCR-2 region.

### Nucleotide sequence accession numbers. 

The nucleotide sequences of TePV1 have been deposited in GenBank under the accession numbers KC460986 and KC460987.
